# Supplementary material for: Impact of Elicitation on Plant Antioxidants Production in Taxus Cell Cultures
Source: Antioxidants (Basel). 2023 Apr 5;12(4):887. doi: 10.3390/antiox12040887 (PMC10135721; doi:10.3390/antiox12040887)
Supplement: Supplementary file 1 [file antioxidants-12-00887-s001.zip › antioxidants-2310771-supplementary.pdf]

Supplementary material.

Impact of Elicitation on Plant Antioxidants Production in Taxus Cell Cultures

Edgar Perez-Matas[1], Pascual Garcia-Perez[2,3], Mercedes Bonfill[1], Luigi Lucini[2], Diego Hidalgo-Martinez[1,*] and Javier Palazon[1,*]

[1] Department of Biology, Healthcare and the Environment, Faculty of Pharmacy and Food Sciences, University of Barcelona, 08028 Barcelona, Spain

[2] Department for Sustainable Food Process, Università Cattolica Del Sacro Cuore, Via Emilia Parmense 84, 29122 Piacenza, Italy

[3] Nutrition and Bromatology Group, Department of Analytical and Food Chemistry, Faculty of Food Science and Technology, Ourense Campus, Universidade de Vigo, 32004 Ourense, Spain

* Correspondence: dhidalgo@ub.edu (D.H.-M.); javierpalazon@ub.edu (J.P.)

Table S1. Phenolic compound list.

Compound	Class	Subclass	Retention Time (min)	Mass	Formula
Cyanidin	Flavonoids	Anthocyanins	1.79	287.05	C15 H11 O6
Cyanidin 3-O-(6''-*p*-coumaroyl-glucoside)	Flavonoids	Anthocyanins	3.71	595.13	C30 H27 O13
(+)-Catechin	Flavonoids	Flavanols	5.37	290.08	C15 H14 O6
(+)-Gallocatechin	Flavonoids	Flavanols	4.20	306.07	C15 H14 O7
Procyanidin dimer B1	Flavonoids	Flavanols	4.56	578.14	C30 H26 O12
Procyanidin trimer C1	Flavonoids	Flavanols	4.58	866.20	C45 H38 O18
Prodelphinidin dimer B3	Flavonoids	Flavanols	3.24	610.12	C30 H26 O14
Prodelphinidin trimer C-GC-C	Flavonoids	Flavanols	3.94	882.19	C45 H38 O19
(+)-Gallocatechin 3-O-gallate	Flavonoids	Flavanols	3.08	458.10	C22 H18 O11
Prodelphinidin trimer GC-GC-C	Flavonoids	Flavanols	3.23	898.18	C45 H38 O20
Butein	Flavonoids	Chalcones	9.56	272.06	C15 H12 O5
Phloretin	Flavonoids	Dihydrochalcones	7.79	274.08	C15 H14 O5
6-Prenylnaringenin	Flavonoids	Flavanones	7.12	340.13	C20 H20 O5
Eriodictyol	Flavonoids	Flavanones	6.92	288.06	C15 H12 O6
Naringenin 7-O-glucoside	Flavonoids	Flavanones	7.88	434.12	C21 H22 O10
Pinocembrin	Flavonoids	Flavanones	6.02	256.07	C15 H12 O4
Apigenin 6-C-glucoside	Flavonoids	Flavones	4.97	432.10	C21 H20 O10
Gardenin B	Flavonoids	Flavones	6.26	358.11	C19 H18 O7
Tetramethylscutellarein	Flavonoids	Flavones	1.25	342.11	C19 H18 O6
6''-O-Acetylglycitin	Flavonoids	Isoflavonoids	7.54	488.12	C24 H24 O11
Xanthohumol	Flavonoids	Chalcones	5.33	354.15	C21 H22 O5
Luteolin 7-O-(2-apiosyl-glucoside)	Flavonoids	Flavones	19.70	580.13	C26 H28 O15
6''-O-Malonyldaidzin	Flavonoids	Isoflavonoids	3.84	502.13	C24 H22 O12
3-Hydroxyphloretin 2'-O-glucoside	Flavonoids	Dihydrochalcones	12.50	452.14	C21 H24 O11
Sinensetin	Flavonoids	Flavones	11.05	372.12	C20 H20 O7
Quercetin 3-O-xylosyl-glucuronide	Flavonoids	Flavonols	2.80	610.12	C26 H26 O17
Dihydroquercetin 3-O-rhamnoside	Flavonoids	Dihydroflavonols	6.91	450.11	C21 H22 O11
Dihydromyricetin 3-O-rhamnoside	Flavonoids	Dihydroflavonols	2.50	466.12	C21 H22 O12
7-Hydroxymatairesinol	Lignans	Lignans	7.12	374.14	C20 H22 O7
Anhydro-secoisolariciresinol	Lignans	Lignans	8.97	344.16	C20 H24 O5
Cyclolariciresinol	Lignans	Lignans	7.86	360.15	C20 H24 O6
Episesaminol	Lignans	Lignans	8.97	370.10	C20 H18 O7
Sesamol	Lignans	Lignans	5.32	138.03	C7 H6 O3
Lariciresinol-sesquilignan	Lignans	Lignans	21.84	556.24	C30 H36 O10
Conidendrin	Lignans	Lignans	9.22	356.12	C20 H20 O6
1-Acetoxypinoresinol	Lignans	Lignans	2.56	416.15	C22 H24 O8
4-Ethylcatechol	Other polyphenols	Alkylphenols	6.54	138.07	C8 H10 O2
Bergapten	Other polyphenols	Furanocoumarins	9.46	216.04	C12 H8 O4
Coumarin	Other polyphenols	Hydroxycoumarins	6.92	146.03	C9 H6 O2
Esculetin	Other polyphenols	Hydroxycoumarins	7.59	178.02	C9 H6 O4
Esculin	Other polyphenols	Hydroxycoumarins	4.65	340.08	C15 H16 O9

Compound	Class	Subclass	Retention Time (min)	Mass	Formula
2-Methoxy-5-prop-1-enylphenol	Other polyphenols	Hydroxyphenylpropenes	1.21	164.09	C10 H12 O2
Anethole	Other polyphenols	Hydroxyphenylpropenes	2.56	148.09	C10 H12 O
[6]-Gingerol	Other polyphenols	Hydroxyphenylpropenes	18.62	300.23	C17 H32 O4
Pyrogallol	Other polyphenols	Other polyphenols	9.50	126.03	C6 H6 O3
Carnosic acid	Other polyphenols	Phenolic terpenes	13.66	332.19	C20 H28 O4
Carnosol	Other polyphenols	Phenolic terpenes	11.38	330.18	C20 H26 O4
Epirosmanol	Other polyphenols	Phenolic terpenes	11.60	346.17	C20 H26 O5
3,4-DHPEA-EDA	Other polyphenols	Tyrosols	16.29	320.13	C17 H20 O6
Hydroxytyrosol	Other polyphenols	Tyrosols	10.02	154.06	C8 H10 O3
Ligstroside	Other polyphenols	Tyrosols	6.93	524.18	C25 H32 O12
Ligstroside-aglycone	Other polyphenols	Tyrosols	14.85	362.14	C19 H22 O7
p-HPEA-AC	Other polyphenols	Tyrosols	2.19	180.08	C10 H12 O3
Carvacrol	Other polyphenols	Phenolic terpenes	8.48	150.10	C10 H14 O
4-Ethylguaiacol	Other polyphenols	Alkylmethoxyphenols	7.05	152.08	C9 H12 O2
4-Vinylguaiacol	Other polyphenols	Alkylmethoxyphenols	4.95	150.07	C9 H10 O2
4-Hydroxycoumarin	Other polyphenols	Hydroxycoumarins	13.48	162.03	C9 H6 O3
3,4-Dihydroxyphenylglycol	Other polyphenols	Other polyphenols	4.98	170.06	C8 H10 O4
Scopoletin	Other polyphenols	Hydroxycoumarins	4.97	192.04	C10 H8 O4
p-Anisaldehyde	Other polyphenols	Hydroxybenzaldehydes	3.17	136.06	C8 H8 O2
Syringaldehyde	Other polyphenols	Hydroxybenzaldehydes	1.21	182.06	C9 H10 O4
4-Hydroxybenzoic acid 4-O-glucoside	Phenolic acids	Hydroxybenzoic acids	5.09	300.08	C13 H16 O8
Vanillic acid	Phenolic acids	Hydroxybenzoic acids	1.07	168.04	C8 H8 O4
3-*p*-Coumaroylquinic acid	Phenolic acids	Hydroxycinnamic acids	4.67	338.09	C16 H18 O8
5-5'-Dehydrodiferulic acid	Phenolic acids	Hydroxycinnamic acids	6.13	386.09	C20 H18 O8
Avenanthramide 2p	Phenolic acids	Hydroxycinnamic acids	7.52	299.08	C16 H13 N O5
Ferulic acid	Phenolic acids	Hydroxycinnamic acids	9.46	194.06	C10 H10 O4
Ferulic acid 4-O-glucoside	Phenolic acids	Hydroxycinnamic acids	7.12	356.11	C16 H20 O9
Feruloyl tartaric acid	Phenolic acids	Hydroxycinnamic acids	2.94	326.06	C14 H14 O9
Hydroxycaffeic acid	Phenolic acids	Hydroxycinnamic acids	1.21	196.04	C9 H8 O5
p-Coumaric acid ethyl ester	Phenolic acids	Hydroxycinnamic acids	7.21	192.07	C11 H12 O3
Rosmarinic acid	Phenolic acids	Hydroxycinnamic acids	1.23	360.09	C18 H16 O8
Sinapic acid	Phenolic acids	Hydroxycinnamic acids	1.72	224.07	C11 H12 O5
Sinapine	Phenolic acids	Hydroxycinnamic acids	16.06	310.17	C16 H24 N O5
4-Hydroxybenzaldehyde	Other polyphenols	Hydroxybenzaldehydes	5.47	122.03	C7 H6 O2
Caffeic acid 4-O-glucoside	Phenolic acids	Hydroxycinnamic acids	5.93	342.09	C15 H18 O9
p-Coumaroyl tartaric acid	Phenolic acids	Hydroxycinnamic acids	1.96	296.05	C13 H12 O8
Sinapaldehyde	Other polyphenols	Hydroxycinnamaldehydes	13.47	208.07	C11 H12 O4
p-Coumaroyl tyrosine	Phenolic acids	Hydroxycinnamic acids	5.93	327.12	C18 H17 N O5
Piceatannol	Stilbenes	Stilbenes	15.19	244.07	C14 H12 O4
d-Viniferin	Stilbenes	Stilbenes	4.35	454.14	C28 H22 O6
Pterostilbene	Stilbenes	Stilbenes	5.08	256.12	C16 H16 O3
Resveratrol 3-O-glucoside	Stilbenes	Stilbenes	11.06	390.13	C20 H22 O8

Table S2. Selected VIP markers at 8 days

Compound Name	Class	Subclass	VIP score	SE	logFC (COR vs Control)	logFC (SA vs Control)
4-Hydroxybenzoic acid 4-O-glucoside	Phenolic acids	Hydroxybenzoic acids	1.37	0.17	-6.95	2.87
Carvacrol	Other polyphenols	Phenolic terpenes	1.36	0.45	0.00	9.39
Piceatannol	Stilbenes	Stilbenes	1.33	0.16	-0.21	-2.49
3-*p*-Coumaroylquinic acid	Phenolic acids	Hydroxycinnamic acids	1.33	0.19	-1.61	-2.91
Carnosic acid	Other polyphenols	Phenolic terpenes	1.30	0.40	4.63	6.26
4-Ethylcatechol	Other polyphenols	Alkylphenols	1.24	0.20	-6.95	-9.39
Cyclolariciresinol	Lignans	Lignans	1.24	0.19	-6.95	-9.39
Butein	Flavonoids	Chalcones	1.11	0.15	-1.91	0.06
Naringenin 7-O-glucoside	Flavonoids	Flavanones	1.10	0.26	-1.58	0.01
[6]-Gingerol	Other polyphenols	Hydroxyphenylpropenes	1.09	0.45	-6.95	0.39
Tetramethylscutellarein	Flavonoids	Flavones	1.08	0.10	-1.58	-0.88
Carnosol	Other polyphenols	Phenolic terpenes	1.08	0.33	-1.06	-0.77
p-HPEA-AC	Other polyphenols	Tyrosols	1.08	0.11	-3.87	0.03
Ligstroside	Other polyphenols	Tyrosols	1.07	0.14	-6.95	-0.04
Rosmarinic acid	Phenolic acids	Hydroxycinnamic acids	1.06	0.16	-6.95	-1.48
Gardenin B	Flavonoids	Flavones	1.05	0.07	1.90	1.29
Coumarin	Other polyphenols	Hydroxycoumarins	1.04	0.17	-6.95	-0.13
Dihydromyricetin 3-O-rhamnoside	Flavonoids	Dihydroflavonols	1.03	0.07	6.95	0.00
1-Acetoxypinoresinol	Lignans	Lignans	1.03	0.09	6.95	0.00
Pinocembrin	Flavonoids	Flavanones	1.03	0.21	0.92	0.63
Caffeic acid 4-O-glucoside	Phenolic acids	Hydroxycinnamic acids	1.03	0.12	6.95	0.00
6-Prenylnaringenin	Flavonoids	Flavanones	1.03	0.08	1.03	0.52
Ferulic acid 4-O-glucoside	Phenolic acids	Hydroxycinnamic acids	1.03	0.08	1.01	0.50
(+)-Gallocatechin 3-O-gallate	Flavonoids	Flavanols	1.03	0.11	6.95	9.39
Ligstroside-aglycone	Other polyphenols	Tyrosols	1.03	0.08	-6.95	-1.02
Sinapic acid	Phenolic acids	Hydroxycinnamic acids	1.02	0.18	-6.95	-1.29
Bergapten	Other polyphenols	Furanocoumarins	1.02	0.05	2.57	1.40
Avenanthramide 2p	Phenolic acids	Hydroxycinnamic acids	1.02	0.10	-2.93	-0.89
Feruloyl tartaric acid	Phenolic acids	Hydroxycinnamic acids	1.01	0.08	4.38	2.80
7-Hydroxymatairesinol	Lignans	Lignans	1.01	0.07	2.66	1.36
Esculin	Other polyphenols	Hydroxycoumarins	1.01	0.13	1.00	-0.09
Episesaminol	Lignans	Lignans	1.01	0.19	0.76	0.33
Anhydro-secoisolariciresinol	Lignans	Lignans	1.00	0.06	2.63	1.14
3-Hydroxyphloretin 2'-O-glucoside	Flavonoids	Dihydrochalcones	1.00	0.26	6.95	0.00

Table S3. Selected VIP markers at 16 days

Compound Name	Class	Subclass	VIP score	SE	logFC (COR vs Control)	logFC (SA vs Control)
Epirosmanol	Other polyphenols	Phenolic terpenes	1.14	0.11	2.94	2.90
Conidendrin	Lignans	Lignans	1.14	0.12	4.00	0.00
1-Acetoxypinoresinol	Lignans	Lignans	1.14	0.13	4.00	0.00
3,4-Dihydroxyphenylglycol	Other polyphenols	Other polyphenols	1.14	0.13	4.00	0.00
Carnosol	Other polyphenols	Phenolic terpenes	1.14	0.11	3.35	1.35
5-5'-Dehydrodiferulic acid	Phenolic acids	Hydroxycinnamic acids	1.13	0.10	3.02	-13.19
p-Coumaric acid ethyl ester	Phenolic acids	Hydroxycinnamic acids	1.09	0.25	-4.00	3.28
Luteolin 7-O-(2-apiosyl-glucoside)	Flavonoids	Flavones	1.09	0.19	-4.00	3.75
Gardenin B	Flavonoids	Flavones	1.08	0.30	-0.68	2.73
Xanthohumol	Flavonoids	Chalcones	1.08	0.19	-4.00	3.68
p-Anisaldehyde	Other polyphenols	Hydroxybenzaldehydes	1.07	0.09	-4.00	4.56
Prodelphinidin dimer B3	Flavonoids	Flavanols	1.07	0.12	-4.00	-13.19
3-*p*-Coumaroylquinic acid	Phenolic acids	Hydroxycinnamic acids	1.07	0.07	-4.00	-13.19
p-Coumaroyl tartaric acid	Phenolic acids	Hydroxycinnamic acids	1.06	0.10	-4.00	-13.19
Sinapic acid	Phenolic acids	Hydroxycinnamic acids	1.05	0.17	-4.00	-13.19
Hydroxytyrosol	Other polyphenols	Tyrosols	1.05	0.16	-0.58	1.44
Butein	Flavonoids	Chalcones	1.04	0.10	-4.00	7.84
Feruloyl tartaric acid	Phenolic acids	Hydroxycinnamic acids	1.04	0.09	-1.27	-13.19
Anethole	Other polyphenols	Hydroxyphenylpropenes	1.04	0.15	2.00	3.94
Esculin	Other polyphenols	Hydroxycoumarins	1.03	0.08	0.94	-13.19
Lariciresinol-sesquilignan	Lignans	Lignans	1.03	0.34	2.26	2.92
(+)-Gallocatechin	Flavonoids	Flavanols	1.03	0.13	-0.94	-13.19
Resveratrol 3-O-glucoside	Stilbenes	Stilbenes	1.03	0.13	0.00	13.19
7-Hydroxymatairesinol	Lignans	Lignans	1.03	0.10	-0.57	-13.19
Esculetin	Other polyphenols	Hydroxycoumarins	1.03	0.10	1.23	5.19
Tetramethylscutellarein	Flavonoids	Flavones	1.03	0.08	0.09	0.72
Episesaminol	Lignans	Lignans	1.03	0.09	0.82	-13.19
Bergapten	Other polyphenols	Furanocoumarins	1.03	0.07	0.90	1.28
Cyclolariciresinol	Lignans	Lignans	1.02	0.41	4.00	0.00
(+)-Catechin	Flavonoids	Flavanols	1.02	0.07	-0.06	-13.19
Hydroxycaffeic acid	Phenolic acids	Hydroxycinnamic acids	1.02	0.22	0.97	3.52
Caffeic acid 4-O-glucoside	Phenolic acids	Hydroxycinnamic acids	1.02	0.08	0.82	-13.19
6-Prenylnaringenin	Flavonoids	Flavanones	1.02	0.08	0.18	-4.78
Ferulic acid 4-O-glucoside	Phenolic acids	Hydroxycinnamic acids	1.02	0.08	0.20	-4.64
Sinensetin	Flavonoids	Flavones	1.02	0.19	0.00	13.19
(+)-Gallocatechin 3-O-gallate	Flavonoids	Flavanols	1.02	0.11	0.77	-13.19
Avenanthramide 2p	Phenolic acids	Hydroxycinnamic acids	1.02	0.09	0.71	-13.19
Syringaldehyde	Other polyphenols	Hydroxybenzaldehydes	1.02	0.23	0.93	3.61
Ferulic acid	Phenolic acids	Hydroxycinnamic acids	1.02	0.06	0.93	1.32
Sesamol	Lignans	Lignans	1.01	0.13	-0.43	-13.19
Anhydro-secoisolariciresinol	Lignans	Lignans	1.00	0.12	0.53	-13.19

Table S4. Selected VIP markers at 24 days

Compound Name	Class	Subclass	VIP score	SE	logFC (COR vs Control)	logFC (SA vs Control)
Esculetin	Other polyphenols	Hydroxycoumarins	1.20	0.10	2.05	1.09
3,4-Dihydroxyphenylglycol	Other polyphenols	Other polyphenols	1.20	0.11	4.21	0.25
Conidendrin	Lignans	Lignans	1.18	0.10	2.98	1.79
Epirosmanol	Other polyphenols	Phenolic terpenes	1.18	0.11	1.09	0.33
Sinapaldehyde	Other polyphenols	Hydroxycinnamaldehydes	1.17	0.11	1.17	-0.38
5-5'-Dehydrodiferulic acid	Phenolic acids	Hydroxycinnamic acids	1.16	0.16	2.23	0.55
Anethole	Other polyphenols	Hydroxyphenylpropenes	1.14	0.13	-1.69	1.32
Scopoletin	Other polyphenols	Hydroxycoumarins	1.14	0.34	6.32	0.00
Carnosic acid	Other polyphenols	Phenolic terpenes	1.11	0.37	1.22	1.37
4-Hydroxycoumarin	Other polyphenols	Hydroxycoumarins	1.10	0.12	1.44	-0.20
3,4-DHPEA-EDA	Other polyphenols	Tyrosols	1.05	0.09	0.27	-8.16
Hydroxycaffeic acid	Phenolic acids	Hydroxycinnamic acids	1.05	0.26	0.86	2.08
Syringaldehyde	Other polyphenols	Hydroxybenzaldehydes	1.04	0.33	0.82	2.12
3-*p*-Coumaroylquinic acid	Phenolic acids	Hydroxycinnamic acids	1.04	0.05	-0.09	-8.16
Bergapten	Other polyphenols	Furanocoumarins	1.03	0.07	-0.02	-1.42
Tetramethylscutellarein	Flavonoids	Flavones	1.03	0.09	-1.89	0.05
Ferulic acid	Phenolic acids	Hydroxycinnamic acids	1.03	0.07	-0.07	-1.43
Feruloyl tartaric acid	Phenolic acids	Hydroxycinnamic acids	1.02	0.08	-6.32	-1.16
Ferulic acid 4-O-glucoside	Phenolic acids	Hydroxycinnamic acids	1.02	0.08	-1.66	-0.21
6-Prenylnaringenin	Flavonoids	Flavanones	1.02	0.08	-1.70	-0.25
7-Hydroxymatairesinol	Lignans	Lignans	1.02	0.08	-6.32	-1.85
Xanthohumol	Flavonoids	Chalcones	1.02	0.63	6.32	0.00
Caffeic acid 4-O-glucoside	Phenolic acids	Hydroxycinnamic acids	1.01	0.08	-6.32	-3.04
Butein	Flavonoids	Chalcones	1.01	0.16	6.32	8.16
4-Hydroxybenzoic acid 4-O-glucoside	Phenolic acids	Hydroxybenzoic acids	1.01	0.09	-6.32	-8.16
p-Coumaroyl tyrosine	Phenolic acids	Hydroxycinnamic acids	1.01	0.09	-6.32	-8.16
Esculin	Other polyphenols	Hydroxycoumarins	1.01	0.09	-2.18	-1.45
(+)-Gallocatechin	Flavonoids	Flavanols	1.01	0.10	-6.32	-8.16
Avenanthramide 2p	Phenolic acids	Hydroxycinnamic acids	1.01	0.10	-3.09	-8.16
Pinocembrin	Flavonoids	Flavanones	1.01	0.11	-6.32	-8.16
Gardenin B	Flavonoids	Flavones	1.01	0.11	-6.32	-8.16
d-Viniferin	Stilbenes	Stilbenes	1.01	0.13	-6.32	-8.16
Episesaminol	Lignans	Lignans	1.01	0.15	-0.46	0.56
Procyanidin dimer B1	Flavonoids	Flavanols	1.00	0.14	-6.32	-8.16
Luteolin 7-O-(2-apiosyl-glucoside)	Flavonoids	Flavones	1.00	0.25	6.32	8.16
Piceatannol	Stilbenes	Stilbenes	1.00	0.12	-0.95	-0.61
